# Benign Transformation of Atypical Meningioma: A Rare Histopathological Phenomenon at Recurrence

**DOI:** 10.1155/cris/4118914

**Published:** 2024-12-01

**Authors:** Rahaf F. Alanazi, Nasser Alharbi, Ali Alkhaibary, Fahd AlSufiani, Ahmed Aloraidi

**Affiliations:** ^1^College of Medicine, King Saud bin Abdulaziz University for Health Sciences, Riyadh, Saudi Arabia; ^2^King Abdullah International Medical Research Center, Riyadh, Saudi Arabia; ^3^Division of Neurosurgery, Department of Surgery, King Abdulaziz Medical City, Ministry of National Guard-Health Affairs, Riyadh, Saudi Arabia; ^4^Department of Medicine, Division of Physical Medicine and Rehabilitation, King Abdulaziz Medical City, Ministry of National Guard-Health Affairs, Riyadh, Saudi Arabia; ^5^Department of Pathology, King Abdulaziz Medical City, Ministry of National Guard-Health Affairs, Riyadh, Saudi Arabia

**Keywords:** atypical, benign, meningiothelial, transformation, WHO grade

## Abstract

**Background and importance:** Meningiomas are one of the most frequent primary central nervous system (CNS) tumors. According to the World Health Organization (WHO) classification of brain tumors, meningiomas are categorized into Grade 1 (benign meningioma; 80%), Grade 2 (atypical meningioma; 4%–15%), and Grade 3 (anaplastic meningiomas; 1%–3%). Grade 2 meningioma has a higher recurrence rate, ranging from 29%–52%. However, the transformation from atypical meningioma into benign meningioma is poorly understood. The present article describes a patient with Grade 2 meningioma that transformed into a benign subtype.

**Case presentation:** A 51-year-old female with a history of seizures, presented with left-sided progressive weakness. Radiological imaging revealed a large extra-axial parasagittal lesion measuring 5 cm × 5 cm × 4.8 cm, suggestive of meningioma. The patient underwent subtotal resection of the lesion. Histologically, the tumor was in favor of Grade 2 meningioma. Radiological follow-up 8 years postoperatively revealed a recurrent meningioma. The patient underwent right-sided craniotomy and resection of the parasagittal meningioma. The histopathological features were suggestive of a Grade 1 meningioma.

**Conclusion:** The transformation from atypical to benign meningiomas is rarely reported and the mechanism remains unclear. The present case provides insights into the natural history of this entity, describes possible etiologies, and lists the surgical management with an emphasis on preoperative radiological imaging and histopathological investigations.

## 1. Introduction

Meningiomas represent approximately 36% of primary central nervous system (CNS) tumors with an annual incidence of 9.45 per 100,000. They are more common in females compared to males [1]. Meningiomas arise from the arachnoid cap cells which can be found on the inner surface of the dura. Additionally, they can be found in the ventricles, extracranially, as well as in the spinal–dural and intracranial surfaces [2]. Meningiomas are categorized according to the World Health Organization (WHO) classifications into three main grades: benign (Grade 1), atypical (Grade 2), and anaplastic (Grade 3) [3].

There is a significantly higher recurrence rate for WHO Grades 2 and 3 meningiomas following both surgical resection and radiotherapy when compared to WHO Grade 1 meningioma. Atypical Grade 2 meningiomas have a recurrence rate ranging from 29% to 52%. Benign transformation of a recurrent atypical meningioma is extremely rare and is poorly understood. The present article describes a patient with Grade 2 meningioma that transformed into a benign subtype.

## 2. Case Presentation

**Figure 1:**
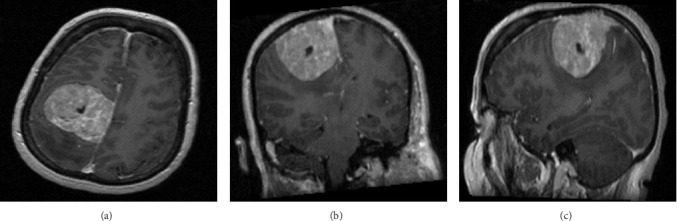
(A–C) Axial, coronal, and sagittal brain MRI post gadolinium administration demonstrating a right parasagittal extra-axial hyper-intense lesion measuring 5 cm × 5 cm × 4.8 cm in AP, transverse, and craniocaudally dimensions, respectively. The lesion is well-defined, oval-shaped, and shows avid enhancement after gadolinium administration. The lesion is causing focal mass effect in the form of compression of the ipsilateral ventricle, midline shift of about 5 mm, and subfalcine herniation. MRI, magnetic resonance imaging.

**Figure 2:**
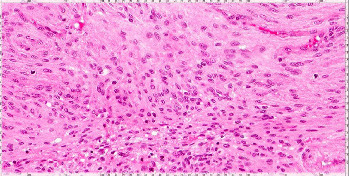
Hematoxylin and eosin stained microscopic image showing meningothelial meningioma with syncytial growth pattern, consisting of bland-appearing meningothelial cells. There is a small pocket of necrosis at the lower middle zone of the picture. Adjacent to the necrotic area, there is increased mitotic figures. Mitotic count in the tumor reaches up to six mitoses per 10 high power fields. These findings are suggestive of meningioma with increased mitotic figures (WHO Grade 2). WHO, World Health Organization.

A 51-year-old female with a history of seizures, presented with left-sided progressive weakness. Radiological imaging showed a large extra-axial lesion measuring 5 cm × 5 cm × 4.8 cm, representing meningioma (Figure 1). The patient underwent presurgical embolization of both middle meningeal arteries with no complications. The tumor was moderately vascular. Afterwards, subtotal resection of the parietal parasagittal meningioma was performed as the tumor was invading the superior sagittal sinus. The histopathological features were suggestive of atypical meningioma; WHO Grade 2 (Figure 2). She was referred to oncology for radiotherapy and was closely followed-up.

**Figure 3:**
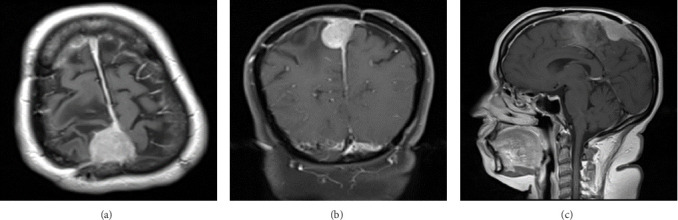
(A–C) Postoperative axial, coronal, and sagittal brain MRI following gadolinium administration. Tumor recurrence is noted at the previous site. It measures 2.8 cm × 2.6 cm × 2.5 cm in three dimensional planes. The tumor is infiltrating the adjacent superior sagittal sinus. MRI, magnetic resonance imaging.

**Figure 4:**
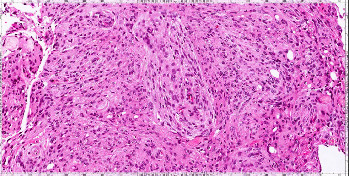
Hematoxylin and eosin stained microscopic image of the recurrent meningioma. The image shows the typical histology of meningothelial meningioma without any atypical features or increased mitotic figures. The findings are keeping up with meningothelial meningioma (WHO Grade 1). WHO, World Health Organization.

Eight years postoperatively, a physical examination revealed that the patient was clinically stable with a Glasgow coma scale of 15/15 and baseline left lower limb weakness (distal > proximal) requiring a walking aid. The left lower foot dorsiflexion was 1/5 (foot drop). The left great toe dorsiflexion was 4/5. Several interval radiological images revealed a slow progression of the residual tumor in the posterior falx. It measured 2.8 cm × 2.6 cm × 2.5 cm, infiltrating the adjacent superior and sagittal sinuses (Figure 3). Considering the clinical and radiological findings, the patient underwent a right-sided craniotomy and resection of the recurrent parasagittal meningioma. The histopathological features were suggestive of a benign typical meningioma (Figure 4).

## 3. Discussion

Meningiomas are typically benign tumors. However, anaplastic and atypical meningiomas are more aggressive, especially anaplastic meningiomas, which are uncommon but are associated with poor prognosis and high recurrence rate [2]. Meningiomas can develop at any area within the skull as they originate from the arachnoid villi [4]. As a result, a wide range of symptoms is usually noted and mainly depends upon the tumor location. These include headache, seizure, hemiparesis, and cranial neuropathies with symptoms such as visual loss [5].

The parasagittal region is the most common location of meningiomas, followed by the flax, cavernous sinus, tuberculum sella, lamina cribrosa, foramen magnum, and torcular zones [6]. Meningiomas are typically located in the skull vault and the skull base. Seventeen percent to twenty percent of all meningiomas are parasagittal, and they most frequently affect the frontal lobe [3].

They can slowly develop to a sizeable lesion over a prolonged period of time and paradoxically remain asymptomatic. Of note, Jacksonian seizures and headache are the most noticeable symptoms of meningioma. Papilledema and homonymous hemianopia could develop if the meningioma was located within the proximity of the optic nerves [7]. The main symptoms at the time of admission for the patient in the present case were headache and blurred vision, which partially matched the statistical characteristics of presentation. The tumor was located in the parietal parasagittal region.

Advanced radiological imaging techniques are crucial for the diagnosis of atypical meningiomas. The most frequently utilized imaging modalities are computed tomography (CT) and magnetic resonance imaging (MRI). On CT and MRI images, meningiomas exhibit two highly distinctive features: a “dural tail” sign that denotes the point at which the tumor and dura are connected, and a “mottling” structure that denotes the high vascularization of the tumor [8]. Occasionally, additional imaging methods are utilized to support the diagnosis. For instance, angiography can be performed to rule out the presence of aneurysms, delineate the tumor relation adjacent to major vessels, or diagnose other vascular lesions.

Although image-based identification is simple and helpful, a histopathologic diagnosis is of utmost importance as meningioma histologic grading carries a prognostic and therapeutic clinical significance. The least common and most aggressive subtype is anaplastic meningioma. Anaplastic meningiomas carries the worst prognosis of the three categories, with an average survival time of less than 2 years [9]. Even benign meningiomas, however, have the potential to histologically advance to cancer; the mechanism behind benign transition is still unknown. According to an earlier study [1, 10, 11], the transition process may take 2–16 years. In the present case, metamorphosis required 8 years to develop.

In the majority of cases, surgical resection is an effective treatment modality for the management of meningioma. Total excision of meningioma provides the best course of management and yields the lowest risk of recurrence. Although total resection is frequently curative, it may not always be possible depending on the lesion's magnitude, location, and bony and dural invasion. The postoperative recurrence rate may dramatically increase with subtotal resection [3]. Radiotherapy, chemotherapy, and recurrent surgical resection are all available treatment options for local recurrence.

Although radical resection carries a substantially higher surgical risk and may render the patient at risk, it can offer a better long-term outcome. When a major resection is difficult due to surgical risks, a partial resection can offer palliative relief. However, the likelihood of recurrence is higher and is mainly dependent upon the extent of resection.

Recurrent meningiomas have been commonly managed using nonsurgical alternatives. Chemotherapy and radiation are the two nonsurgical treatment modalities most frequently utilized. However, the use of radiotherapy and chemotherapy in therapeutic management is not universally agreed upon. Radiation therapy is thought to be required for WHO Grade 3 meningiomas due to their propensity for recurrence and aggressive behavior. Chemotherapy is reserved for recurring meningiomas after all other treatments have failed because it has not been proven to be effective against atypical and anaplastic meningiomas [12, 13].

Meningioma metastasis is not prevalent. Metastases are uncommon, even when they have benign histological characteristics [10]. Infiltration and fast growth are more frequent manifestations of benign histology, which raises the possibility of local recurrences after surgical removal. However, metastasis can also spread through the blood, lymph, or cerebrospinal fluid; dissemination during surgery is not one of them. Meningioma metastasis most frequently occurs in the dura, brain, and lung. Unfortunately, no trustworthy indicators of meningioma metastases have been discovered.

Most reported metastatic meningiomas at the time of presentation have benign histological features. Only a small number of atypical occurrences have, thus far, been documented [11, 14, 15]. In the present case, it is intriguing to note that the histopathological features were initially suggestive of atypical meningioma (WHO Grade 2). However, 8 years postoperatively, the patient had recurrence of meningioma. A surgical resection of the lesion was performed. The histopathological features were diagnostic of a benign meningioma (WHO Grade 1). The benign transformation from atypical to typical meningioma was established based on meticulous histopathological evaluation. The histopathological diagnosis was carefully reviewed and confirmed by a neuropathologist. Maiuria et al. [16] theorized that atypical meningiomas may harbor nests of benign tumor cells peripheral to the initial tumor location. Therefore, explaining the discrepancies of histopathological diagnosis between initial and recurrent meningioma [16].

## 4. Conclusion

WHO Grades 2 and 3 meningiomas are associated with a much greater risk of recurrence following both surgical resection and radiotherapy than WHO Grade 1 meningiomas. Even WHO Grade 1 meningiomas can infrequently recur and cause neurological sequelae. Patients with recurrent meningiomas may require multiple and repeated surgical resections. The likelihood of recovery is greatly decreased when multiple operations are performed. Rarely, an atypical meningioma may undergo a histological benign transformation. The mechanism of transformation remains uncertain. Further studies are required to investigate, suggest, and hypothesize possible theories of meningiomas histologically transforming into lower grades.
